# Supplementary figures and images for: Effects of Varied Stimuli on Escape Behavior Diversification of Himalayan Marmots for Different Human Disturbances
Source: Animals (Basel). 2025 Mar 25;15(7):935. doi: 10.3390/ani15070935 (PMC11988062; doi:10.3390/ani15070935)

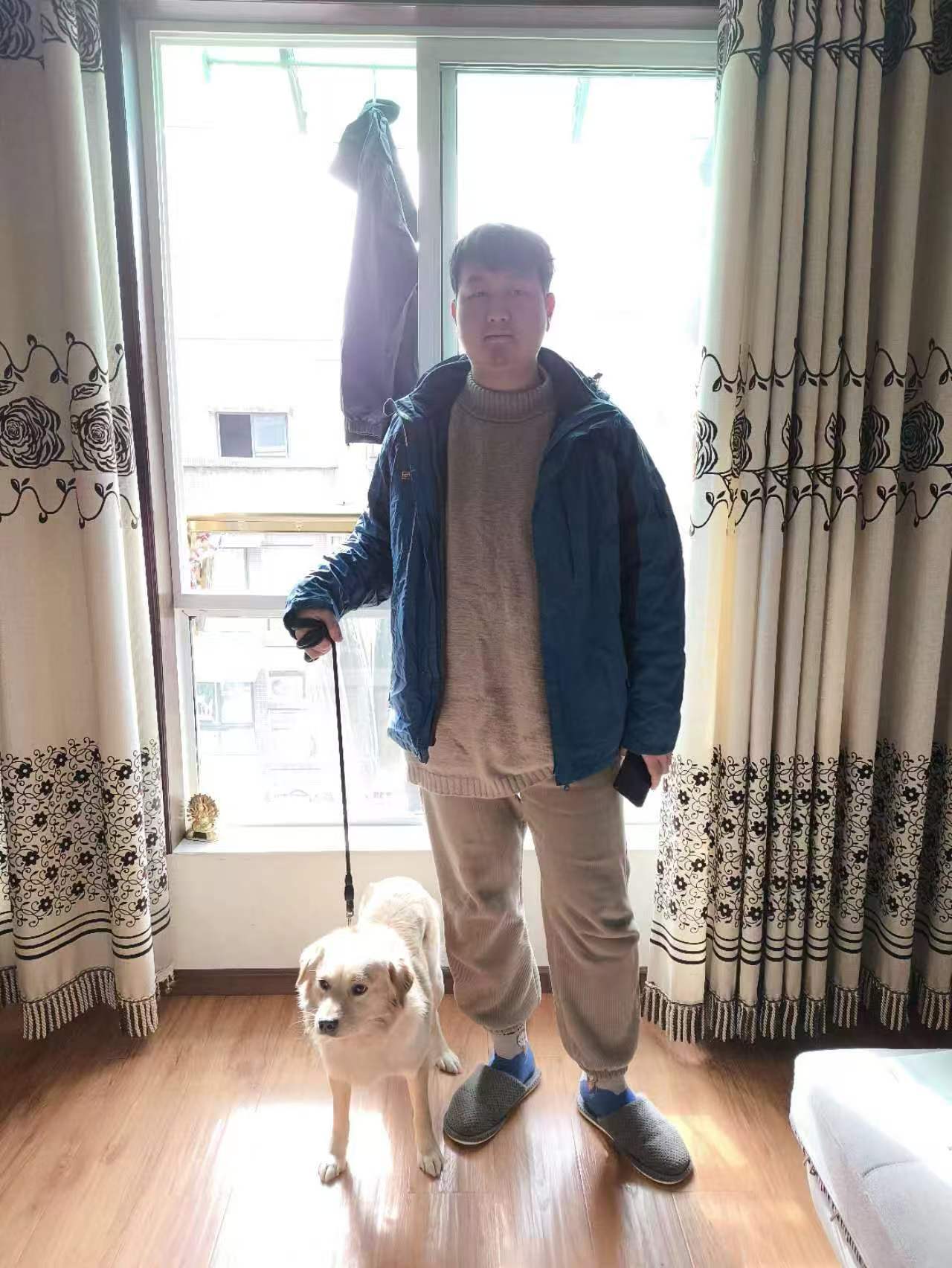

Supplement: Supplementary file 1 [file animals-15-00935-s001.zip › Figure S1.jpg]
